# A Case of Nevus of Ota in Trinidad and Tobago

**DOI:** 10.7759/cureus.78311

**Published:** 2025-01-31

**Authors:** Rajiv V Seemongal-Dass, Robin R Seemongal-Dass, Alyssa P Singh, Diego A Conocchiari

**Affiliations:** 1 Ophthalmology, Eyenet Ltd, Chaguanas, TTO

**Keywords:** dermal melanocytosis, nevus of ota, oculodermal melanocytosis, oculodermal melanosis, scleral hyperpigmentation

## Abstract

Oculodermal melanocytosis, also known as Nevus of Ota, was extensively described in 1939 and is characterized by unilateral, irregular, bluish-black cutaneous pigmentation along the distribution of the ophthalmic and maxillary branches of the trigeminal nerve. This condition is non-hereditary and occurs more commonly in females. To the best of our knowledge, we report the first documented case of Nevus of Ota in Trinidad and Tobago, presenting in a 67-year-old female with characteristic pigmentation and no family history of similar conditions. The diagnosis was confirmed clinically, and the patient was counseled on potential ocular and dermatological complications. This case highlights the importance of recognizing rare dermatological conditions in diverse populations to facilitate timely diagnosis, appropriate management, and effective monitoring for potential complications.

## Introduction

Nevus of Ota, also known as oculomucodermal melanocytosis, is a benign congenital hyperpigmentation of the facial skin and mucous membranes, following the distribution of the ophthalmic and maxillary divisions of the trigeminal nerve [1]. In 1939, Dr. Masao Ota provided the first detailed description of its classical clinical presentation, characterized by hyperpigmentation along the cutaneous distribution of the first and second divisions of the trigeminal nerve, often accompanied by mucosal involvement [2].

This condition is very rare, with a reported prevalence of 0.014%-0.034% in the Asian population [3]. While it is most common among individuals of Japanese descent, it has also been documented in those of African, Chinese, Indian, and European ancestry. There is a greater female predominance, with a female-to-male ratio of 5:1 [4].

To the best of our knowledge, this is the first documented case of Nevus of Ota in Trinidad, occurring in a female of East Indian descent. Documenting such cases in diverse populations, including the Caribbean region, is essential for improving global understanding of its presentation, prevalence, and potential genetic or environmental influences. As Trinidad is a multiethnic society with a genetic heritage from Africa, India, China, South America, and other regions, a myriad of factors may contribute to disease patterns, none of which can be isolated. This unique case, however, could help raise ophthalmological awareness of this diagnosis in other patients, paving the way for further assessment of regional and genetic influences on its presentation.

## Case presentation

A 67-year-old female of East Indian descent presented to the ophthalmology clinic for a routine check-up with a history of left keratitis previously treated elsewhere. At presentation, she was asymptomatic, reporting no visual impairment, hearing loss, or sensory deficits. Examination revealed congenital, non-hereditary, unilateral left facial pigmentation with irregular borders that had progressively darkened with age. At this time, she had not previously been provided with an explanation for the hyperpigmentation. Written consent was obtained from the patient for the documentation and discussion of this case.

**Figure 1:**
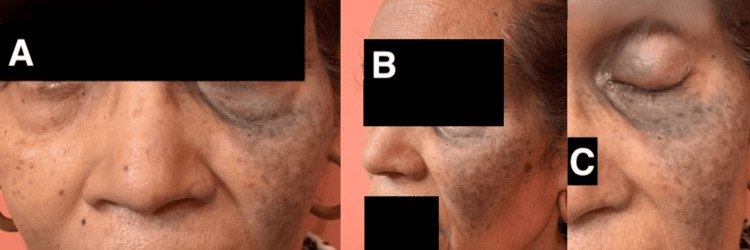
Multiple pigmented regions at presentation A: Cutaneous bluish-black pigmentation on the left side of the face compared to the right side; B: Pigmentation of the left ala of nose and malar region of the face; C: Pigmentation of the left upper eyelid

On cutaneous examination, there were multiple mottled bluish-black macular pigmented regions involving the left malar region, left upper eyelid, left ala of the nose, and the inferonasal sclera of the left eye (Figure 1). There were no pigmentary changes on other parts of the body, including nails, mucous membranes, or axillary regions. 

The patient was hypermetropic and used corrective lenses. Objective refraction revealed +3.00/-1.50 x 120 in the right eye and +3.75/-1.50 x 90 in the left eye. Aided visual acuity was 6/7.5 in the right eye and 6/9-2 in the left eye. Near vision was N5 with +2.50 readers. Intraocular pressures were within normal limits: OD 12 mmHg and OS 11 mmHg, respectively.

The patient had a history of left herpetic keratitis, a 10-year history of hypertension controlled with Atenolol, an eight-year history of diabetes managed with Metformin, and a history of cervical cancer at age 48, treated with chemotherapy and radiation. There have been no further recurrences or complications since treatment. 

**Figure 2:**
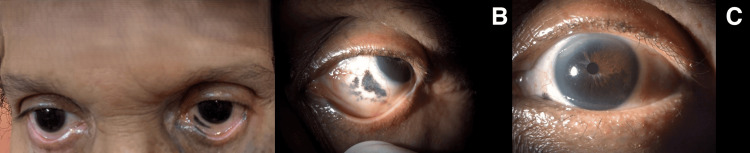
Pigmentation of the left inferonasal part of the sclera with heterochromia iridum A: Pigmentation of the left inferonasal aspect of sclera; B: Left Inferonasal pigmentation; C: Left heterochromia iridum

Anterior segment examination showed left pigment dispersion, left inferonasal pigmentation of the sclera, and left iridum heterochromia secondary to a hyperpigmented iris (Figure 2). Cataracts were present bilaterally. Gonioscopy revealed that the left angle was more heavily pigmented compared to the right, and the hyperpigmented iris was thicker than the normally pigmented one.

Several diagnostic tests were performed. Topography and biometry were done for axial length, anterior chamber depth, and white-to-white measurements. The left eye exhibited a shorter axial length, a shallower anterior chamber, and a smaller white-to-white measurement compared to the right eye (Table 1). 

**Table 1:** Topographic measurements in the years 2019, 2022, and 2024

Measurements	2019	2019	2022	2022	2024	2024
Axial Length (mm)	OD: 22.46	OS: 22.27	OD: 22.45	OS: 22.27	OD:23.39	OS: 22.21
Anterior Chamber Depth (mm)	OD: 3.02	OS: 2.98	OD: 3.01	OS: 2.93	OD: 2.94	OS: 2.89
White-to-White (mm)	OD: 11.55	OS: 11.34	OD: 11.49	OS: 11.34	OD: 11.52	OS: 11.38

**Figure 3:**
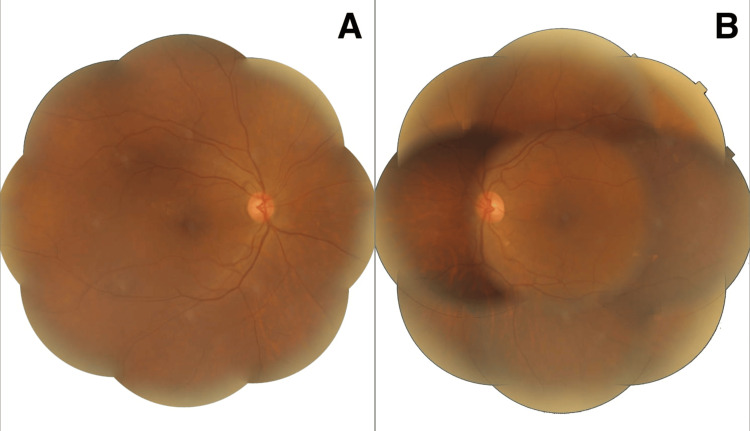
Mosaic of the retina in right and left eyes A: Mosaic of right eye with no abnormalities detected; B: Mosaic of left eye with no abnormalities detected

Optical coherence tomography (OCT) imaging indicated normal retinal structure in both eyes, with no retinal pigmentation detected. Fundus and mosaic imaging showed no abnormalities (Figure 3). Enhanced depth imaging optical coherence tomography (EDI-OCT) B-scan measured the choroidal thickness at 307 µm in the right eye (OD) and 309 µm in the left eye (OS), both within normal parameters. 

**Figure 4:**
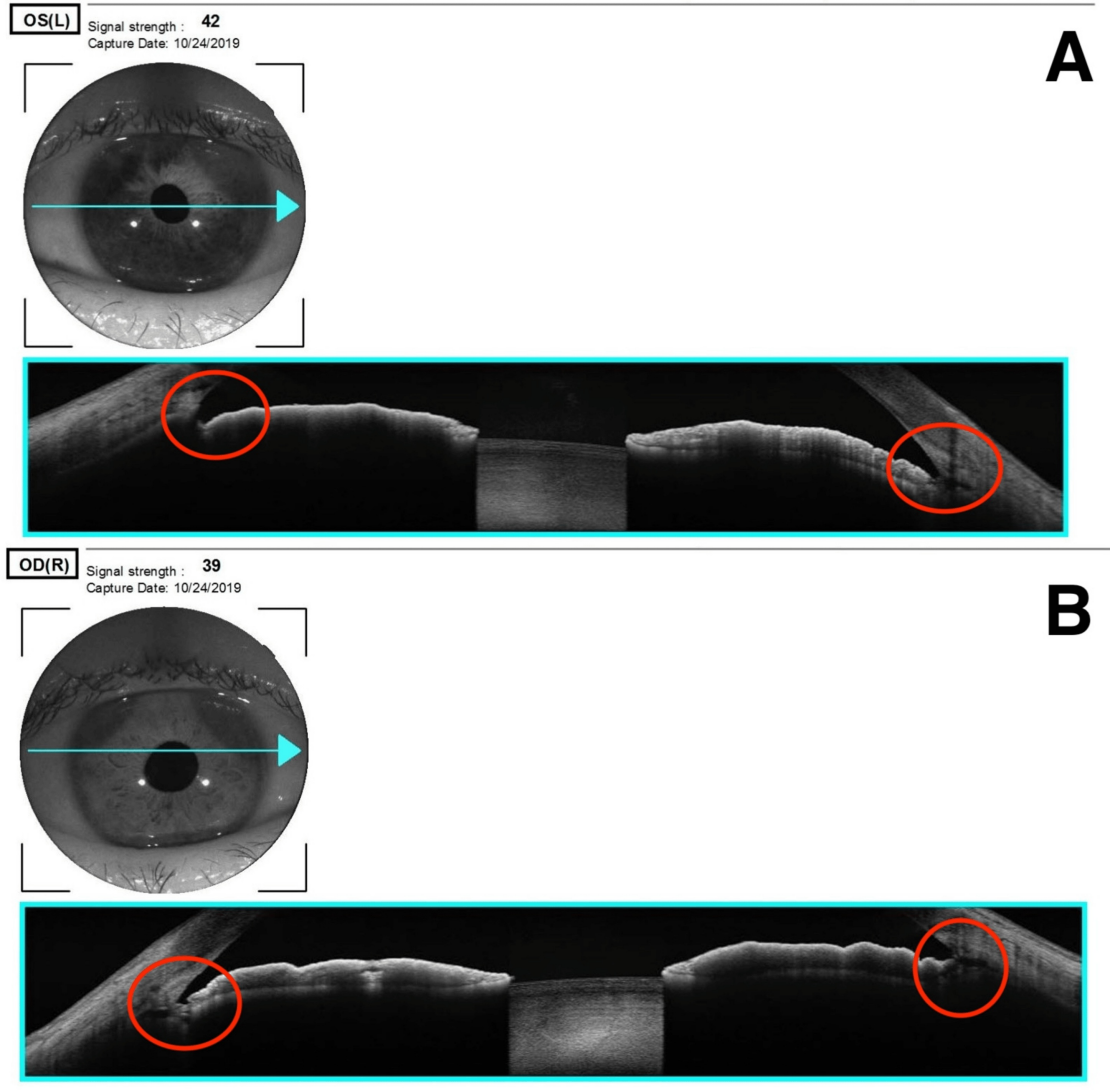
Anterior segment OCT of the angles A: OCT angles of right eye with red circles indicating the angles; B: OCT angles of left eye with red circles indicating the angles OCT, optical coherence tomography

Gonioscopy and OCT angles demonstrated progressive narrowing of the angles in the left eye, indicating a potential risk for pigmentary or narrow-angle glaucoma (Figure 4).

Based on the location, distribution, and characteristic features, a diagnosis of Nevus of Ota was made. The patient remains under regular follow-up due to her increased risk of developing pigmentary glaucoma and the potential need for cataract surgery. Most recently, OCT angle imaging revealed further narrowing of the angles in the left eye, warranting close observation. Thus far, all parameters mentioned previously have remained stable under monitoring. Given her diagnosis of Nevus of Ota, she will continue to be closely observed for any future changes.

## Discussion

Nevus of Ota typically presents as an ipsilateral blue or grey irregular macular patch along the first and second divisions of the trigeminal nerve on the facial skin. It can be congenital, present at birth, or acquired later in life, with acquired cases commonly occurring during the second year of life, puberty, or pregnancy [1,4]. The macular discolorations typically appear as unilateral, confluent, non-hairy, flat-pigmented lesions with poorly defined margins, although bilateral cases can also occur [4]. The characteristic hyperpigmented patches are thought to result from incomplete migration of melanocytes from the neural crest to the epidermis during embryonic development, leading to dermal nesting and melanin production [4]. The pathogenesis of the nevus is multifactorial, with key predisposing factors including genetics, family sex hormones, infections, trauma, and ultraviolet light exposure [4,5].

Pigmentation generally affects the forehead, temple, malar, auricular, nasal, and periorbital regions. Tanino (1939) classified pigmentation into four types based on its distribution [2]. The four categories are based on the area of involvement (Table 2). 

**Table 2:** Tanino's classification of Nevus of Ota

Type	Subtypes	Area of Involvement
Type I	Type IA	Mild Orbital - upper and lower eyelids, periorbital, and temple regions are affected.
Type I	Type IB	Mild Zygomatic - infrapalpebral fold, nasolabial fold, and zygomatic region affected.
Type I	Type IC	Mild Forehead - only the forehead.
Type I	Type ID	Ala nasi only affected.
Type II		Moderate type - upper and lower eyelids, periocular, zygomatic, buccal, and temple regions.
Type III		Distributed over the scalp, forehead, eyebrows, and nose.
Type IV		Bilateral distribution

The case reported here is classified as Tanino type II. The ocular adnexa and globe may also be involved, including the sclera, conjunctiva, cornea, iris, choroid, and, less commonly, the optic nerve, retrobulbar area, and extraocular muscles [5]. The sclera is most frequently affected in patients with Nevus of Ota. Ocular complications associated with Nevus of Ota include glaucoma, cataracts, and choroidal melanoma [6]. Severe pigmentation in the anterior chamber can block the iridocorneal angle, leading to increased intraocular pressure. Nevus of Ota can, therefore, be a cause of secondary glaucoma, and regular ophthalmic examinations are necessary to assess the anterior chamber and intraocular pressure for signs of glaucoma.

Diagnosis of Nevus of Ota is made based on classical clinical features. Differential diagnoses include café-au-lait patches, melasma, and drug-induced hyperpigmentation [7,8]. The pigmentation can be an isolated occurrence or part of a systemic condition, such as Sturge-Weber syndrome, Klippel-Trenaunay syndrome, or neurofibromatosis [7]. A skin biopsy is warranted if the pigmented lesions become ulcerated or change color.

The diagnosis was confirmed by an experienced ophthalmologist based on the clinical presentation. Treatment often involves modern technologies such as YAG laser, Q-switched ruby, and Q-switched alexandrite lasers [9]. The Q-switched Nd:YAG lasers and Q-switched ruby lasers target the melanocytes and melanosomes responsible for the pigmented lesions [10]. These laser treatments have been shown to remove the pigmented lesions without scarring. Dermatological treatments include dermabrasion, epidermal peeling, and argon laser therapy [9]. 

Beyond cutaneous involvement, ocular manifestations of Nevus of Ota, such as choroidal melanocytosis, require careful evaluation [7]. Choroidal melanocytosis, a form of ocular melanocytosis, presents as diffuse uveal hyperpigmentation and is commonly associated with Nevus of Ota. While benign, it significantly increases the risk of uveal melanoma, necessitating lifelong ophthalmic surveillance, including dilated fundus exams, ultrasonography, and OCT for early detection.

## Conclusions

In conclusion, Nevus of Ota is a pigmentary condition that typically presents with characteristic macular patches along the trigeminal nerve distribution on the face, with potential ocular involvement, especially in the sclera. Its pathogenesis is linked to incomplete melanocyte migration during embryonic development, and it can be congenital or acquired, often triggered by factors such as hormonal changes or ultraviolet exposure. While the condition is generally benign, ocular complications such as glaucoma, cataracts, and malignancy may arise making regular ophthalmic monitoring essential. Early diagnosis is primarily clinical, with differential diagnoses including café-au-lait patches and melasma. Treatment options are based on the specific findings, but for the pigmented lesions, advanced laser technologies such as Q-switched Nd:YAG and ruby lasers have shown promising results in effectively removing them while minimizing the risk of scarring. Therefore, a thorough understanding of the clinical features and potential ocular risks is crucial for effective management and prevention of complications in patients with Nevus of Ota. This case highlights the importance for ophthalmologists in the Caribbean region to consider this condition, given the diverse population with mixed genetic backgrounds from across the globe.

## References

[1] Mohan RP, Verma S, Singh AK, Singh U (2013). 'Nevi of Ota: the unusual birthmarks': a case review. BMJ Case Rep.

[2] Bohra A, Bhateja S (2015). "Nevus of Ota": a rare oro-facial pigmentation - short review. J Pigment Disord.

[3] Magarasevic L, Abazi Z (2013). Unilateral open-angle glaucoma associated with the ipsilateral nevus of ota. Case Rep Ophthalmol Med.

[4] Agarwal P, Patel BC (2025). Nevus of Ota and Ito. https://www.ncbi.nlm.nih.gov/books/NBK560574/.

[5] Sayed-Ahmed I, Murillo JC, Monsalve P (2018). Blue nevi of the ocular surface: clinical characteristics, pathologic features, and clinical course. Ophthalmology.

[6] Shields CL, Kaliki S, Livesey M (2013). Association of ocular and oculodermal melanocytosis with the rate of uveal melanoma metastasis: analysis of 7872 consecutive eyes. JAMA Ophthalmol.

[7] Plateroti AM, Scavella V, Abdolrahimzadeh B, Plateroti R, Rahimi S (2017). An update on Oculodermal melanocytosis and rare associated conditions. Semin Ophthalmol.

[8] Radhadevi CV, Charles KS, Lathika VK (2013). Orbital malignant melanoma associated with nevus of Ota. Indian J Ophthalmol.

[9] Yang J, Luo G, Tuyana S, Tong X, Tu Y, Tao J (2016). Analysis of 28 Chinese cases of bilateral nevus of Ota and therapeutic results with the Q-switched alexandrite laser. Dermatol Surg.

[10] Shah VV, Bray FN, Aldahan AS, Mlacker S, Nouri K (2016). Lasers and nevus of Ota: a comprehensive review. Lasers Med Sci.

